# Effect of mitochondrial fission inhibition on C2C12 differentiation

**DOI:** 10.1016/j.dib.2016.02.070

**Published:** 2016-03-05

**Authors:** Darin Bloemberg, Joe Quadrilatero

**Affiliations:** Department of Kinesiology, University of Waterloo, Waterloo, Ontario, Canada

**Keywords:** Skeletal muscle, Mitochondrial fission, Apoptosis, Differentiation, Caspases, C2C12

## Abstract

The differentiation of skeletal muscle is commonly examined in cell culture using the C2C12 line of mouse skeletal myoblasts. This process shares many similarities with that which occurs during embryonic development, such as the transient activation of caspases. Here, we examined the effect of inhibiting mitochondrial fission, using mdivi-1, on the ability of C2C12 cells to terminally differentiate. This was performed using immunofluorescent identification of cell morphology and myosin expression, as well as immunoblotting for markers of muscle differentiation. Furthermore, the effect of mdivi-1 administration on activation of caspase-2 and -3 was assessed using spectrofluorometric measurement of specific enzyme activity.

## Specification Table

**Table:** 

Subject area	*Biology*
More specific subject area	*Skeletal muscle differentiation, caspases, mitochondrial fission*
Type of data	*Graphs and Figures*
How data was acquired	*Fluorescent microscopy, spectrofluorometry, immunoblotting*
Data format	*Analyzed*
Experimental factors	*C2C12 cells were differentiated with the inhibitor of mitochondrial fission, mdivi-1*
Experimental features	*Cells were differentiated while being exposed to different concentrations of mdivi-1 and collected at various time points during the differentiation process. They were then prepared for spectrofluorometric measurement of caspase activity or were assessed for the markers of muscle differentiation, myosin and myogenin, using immunoblotting. Separate cells were immunostained for myosin and imaged using fluorescent microscopy to evaluate changes to cell morphology.*
Data source location	*University of Waterloo, Waterloo, Ontario, Canada*
Data accessibility	*All data are provided with this article*

## Value of the data

• The data demonstrate that myogenic differentiation is prevented by administering a chemical inhibitor of mitochondrial fission.
• Although mitochondrial fission has complex roles regarding the regulation of apoptotic signaling, its inhibition led to significant elevations in caspase activity in this context.
• These data provide evidence that proper mitochondrial fission is important for the dramatic changes that accompany cellular adaptations, and support the execution of further studies in this regard.

## 1 Data

Here, we present data regarding the effect of mitochondrial fission inhibition on skeletal muscle differentiation. Although similar experiments have been published by others [1], [2], the data presented here support and add to the conclusions made by these researchers. Of note, the relatively higher concentration of mdivi-1 utilized here demonstrates the dose-dependence of its effect on C2C12 differentiation [1]. We additionally include data characterizing the activity of caspase-2 during this process.

### 1.1 mdivi-1 administration prevents terminal differentiation of C2C12 cells

**Fig. 1:**
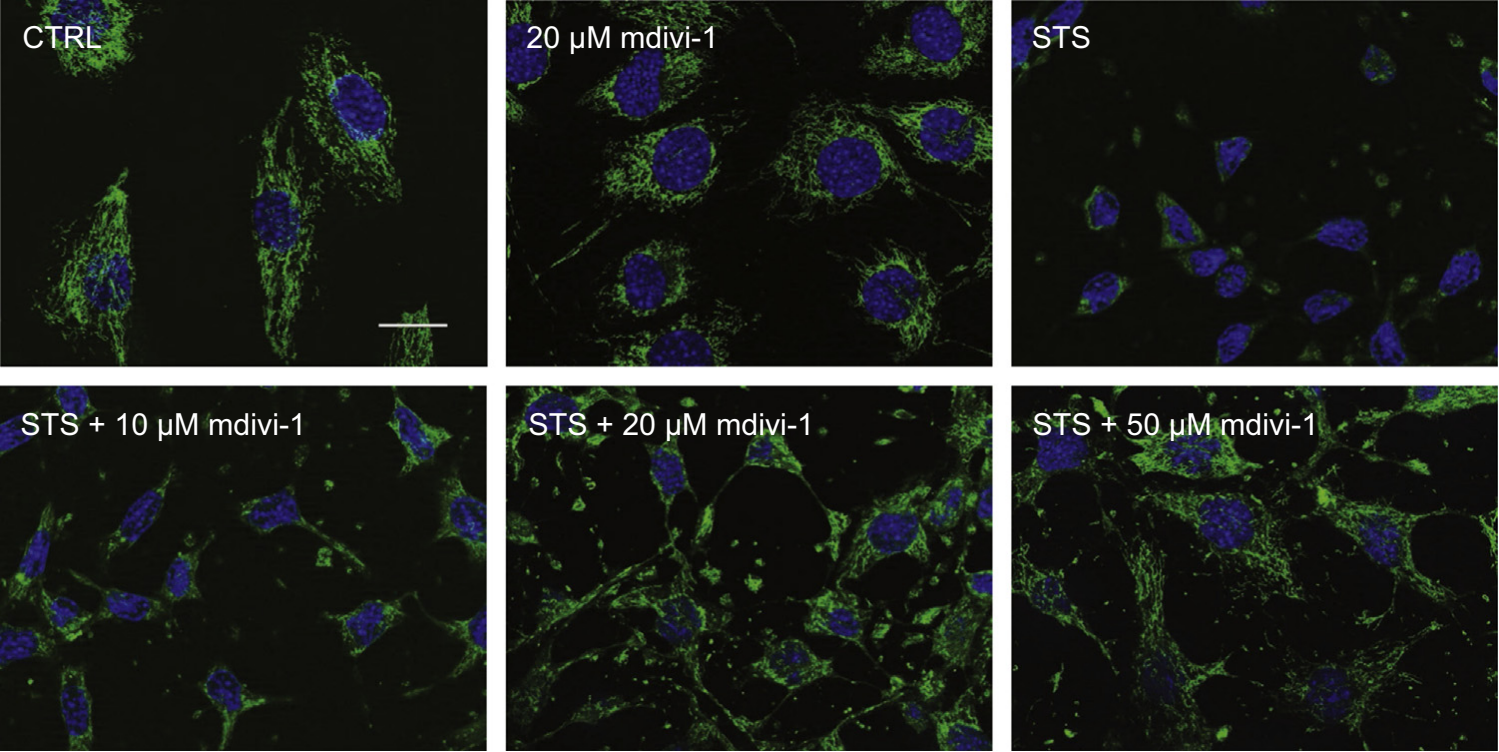
Determination of working mdivi-1 concentrations. Increasing concentrations of mdivi-1 progressively inhibited apoptosis-associated changes in cell morphology induced by 2 h of 2 µM STS. Mitochondria were visualized using MitoTracker (green) and nuclei with DAPI (blue). Scale bar represents 20 µm.

**Fig. 2:**
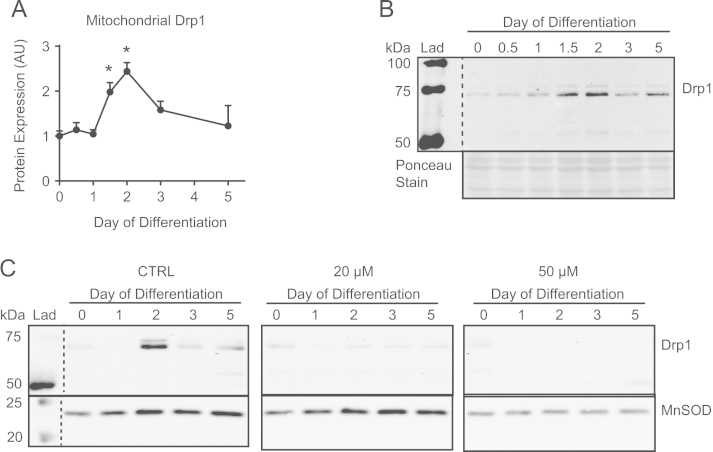
mdivi-1 administration during C2C12 differentiation prevents mitochondrial localization of Drp1. (A and B) C2C12 cells were differentiated and collected at various time points, following which mitochondrial-enriched fractions were immunoblotted for Drp1. (A) Quantification of mitochondrial Drp1 protein demonstrating increased levels on day 1.5 and day 2 compared to day 0. (B) Representative immunoblot of mitochondrial Drp1 and Ponceau staining. (C) C2C12 cells were differentiated in media containing the indicated concentrations of mdivi-1 (20 µM or 50 µM) or DMSO (CTRL), and mitochondrial-enriched subcellular fractions were immunoblotted for Drp1 and the mitochondrial protein MnSOD. ^⁎^*p*<0.05 compared to day 0.

**Fig. 3:**
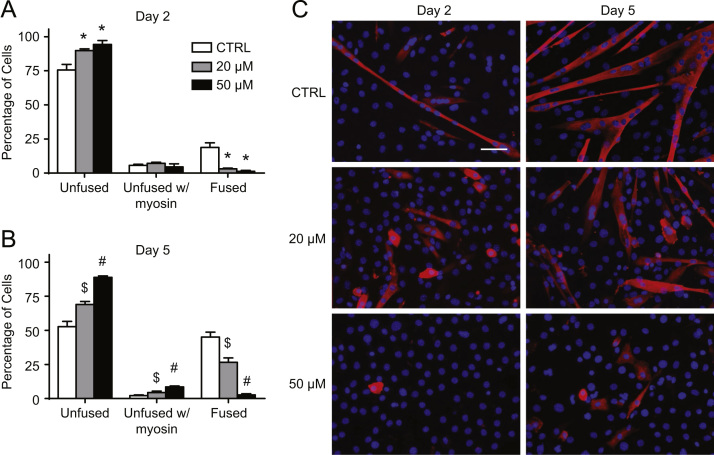
Inhibiting mitochondrial fission impaired cell fusion and myotube formation during C2C12 differentiation. Significantly less nuclei (blue) were contained in multi-nucleated cells displaying myosin (red) on day 2 (A and C) and day 5 (B and C) of differentiation during mdivi-1 administration. ^⁎^*p*<0.05 compared to CTRL, $*p*<0.05 compared to CTRL and 50 µM, # #*p*<0.05 compared to CTRL and 20 µM. Scale bar represents 50 µm.

**Fig. 4:**
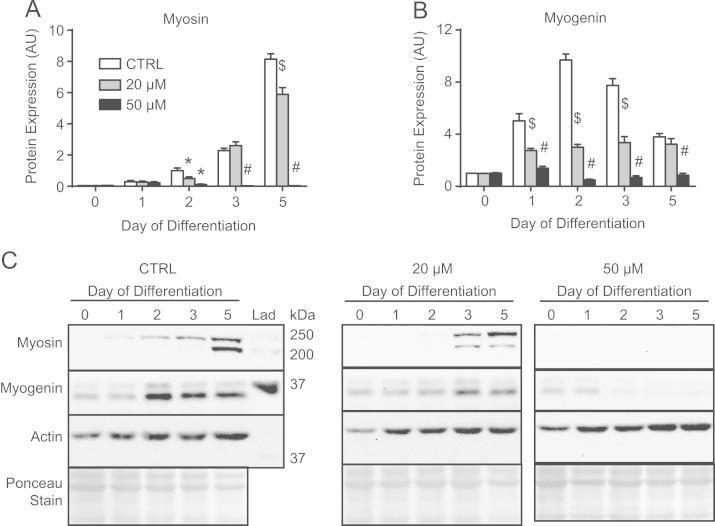
Inhibiting mitochondrial fission during differentiation prevented the increase in markers of skeletal muscle development. (A) Myosin content was decreased on day 5 in cells receiving 20 µM mdivi-1, while its expression was completely abolished by treatment with 50 µM mdivi-1. (B) Similarly, myogenin expression was decreased in cells treated with mdivi-1. (C) Representative immunoblots. ^⁎^*p*<0.05 compared to CTRL, $*p*<0.05 compared to CTRL and 50 µM, #*p*<0.05 compared to CTRL and 20 µM.

**Fig. 5:**
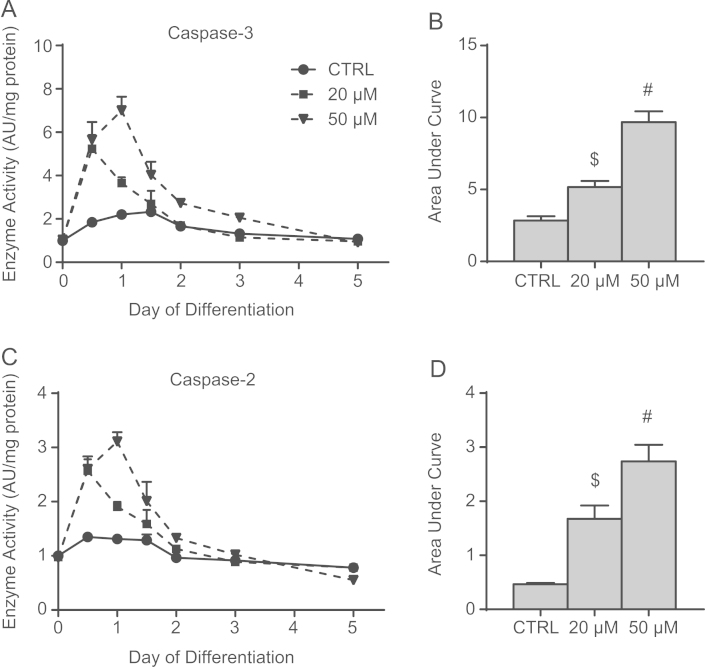
Inhibiting mitochondrial fission increases caspase activity during differentiation. (A) Time course of caspase-3 activity during differentiation. (B) Total amount of caspase-3 activity during differentiation, as determined by area under the curve calculations, was increased by mdivi-1 administration. (C) Time course of caspase-2 activity during differentiation. (D) Total amount of caspase-2 activity during differentiation, as determined by area under the curve calculations, was increased by mdivi-1 administration. Values in (A) and (C) are presented relative to CTRL, given a value of 1.0. $*p*<0.05 compared to CTRL and 50 µM, #*p*<0.05 compared to CTRL and 20 µM.

As was performed previously [3], we began by determining a concentration of mdivi-1 that would maintain mitochondrial and cellular morphology during staurosporine (STS) treatment. Subconfluent C2C12 cells were administered 2 µM STS for 2 h alone or with increasing concentrations of mdivi-1, and mitochondria and nuclei were visualized using MitoTracker Green and DAPI, respectively (Fig. 1). As can be seen, STS induced nuclear condensation and morphological alterations typical of apoptotic cell death, but these changes were progressively prevented by increasing concentrations of mdivi-1 from 10 µM to 50 µM (Fig. 1). As a result, doses of 20 µM and 50 µM mdivi-1 were chosen to conduct C2C12 differentiation experiments. First, we confirmed the alteration of mitochondrial fission signaling during differentiation by immunoblotting the fission mediator Drp1 in mitochondrial-enriched subcellular fractions (Fig. 2A and B). Here, data shows mitochondrial Drp1 content was increased (*p*<0.05) on days 1.5 and 2 compared to day 0 (Fig. 2A and B). Subsequently, C2C12 cells were differentiated in media containing either mdivi-1 (20 µM or 50 µM) or DMSO (CTRL) and collected at various time points during the differentiation process (Figs. 2C, 3–5). Immunoblotting of Drp1 in mitochondrial-enriched fractions demonstrated that 20 µM and 50 µM mdivi-1 decreased mitochondrial localization of Drp1 during differentiation (Fig. 2C). Next, our data demonstrates that inhibiting mitochondrial fission during differentiation prevents myotube formation in a concentration-dependent manner (Fig. 3). In fact, daily treatment with 50 µM mdivi-1 completely impaired myoblast fusion, as indicated by reduced (*p*<0.05) multi-nucleated and myosin positive cells compared to the CTRL condition by day 5 (Fig. 3B and C). Assessment of mature skeletal muscle markers using immunoblotting displayed similar results. While total myosin content and the appearance of myogenin were blunted (*p*<0.05) by 20 µM mdivi-1, their expression was completely abolished (*p*<0.05) by treatment with 50 µM (Fig. 4).

### 1.2 mdivi-1 administration caused dramatic, but transient, activation of caspases during differentiation

Despite ours’ and others’ observations that mdivi-1 can prevent STS-induced apoptotic changes in cell morphology during short term treatment, its administration during C2C12 differentiation resulted in dramatically increased (*p*<0.05) activity of caspase-3 and caspase-2 (Fig. 5B and D). These data show that after only 12 h, caspase-3 activity was 3-fold higher in both mdivi-1 treated groups compared to CTRL, an effect that remained after 24 h of differentiation in the cells given 50 µM (Fig. 5A). Similar effects were observed with caspase-2 (Fig. 5C). Curiously, these effects were transient, as the activity of both caspase-2 and caspase-3 in mdivi-1-treated cells returned to the levels observed in CTRL cells by day 2 (Fig. 5A and C). Nonetheless, it is likely that this temporary dramatic elevation in caspase activity is responsible for the observed reduction in myogenesis [4], [5].

## 2 Experimental design, materials and methods

### 2.1 Cell culture and experiment

C2C12 mouse skeletal myoblasts (ATCC) were cultured on polystyrene cell culture plates (BD Biosciences) with growth media (GM) consisting of low-glucose DMEM (ThermoFisher) with 10% fetal bovine serum (FBS; ThermoFisher) and 1% penicillin/streptomycin (ThermoFisher) [4]. Upon reaching 80–90% confluence, cells were induced to differentiate by replacing GM with differentiation media (DM) consisting of DMEM with 2% horse serum (ThermoFisher). Cells were collected and used for various analyses immediately prior to differentiation (day 0), and at several time points thereafter. In order to prevent mitochondrial fission, the small chemical inhibitor of the mitochondrial division dynamin, mdivi-1 [3] (Enzo Life Sciences), was added to DM at two concentrations: 20 µM and 50 µM. DM was replaced daily, with or without mdivi-1, after washing cells with warm PBS.

### 2.2 Preparation of cell lysates

At the indicated time points, cells were collected via trypsinization, centrifuged at 1000*g*, and stored at −80 °C. Cells were prepared for spectrofluorometry analyses by sonicating cells in lysis buffer containing 20 mM HEPES, 10 mM NaCl, 1.5 mM MgCl, 1 mM DTT, 20% glycerol, and 0.1% Triton-X100 at a pH of 7.4. Immunoblotting samples were sonicated in lysis buffer containing protease inhibitors (Complete Cocktail, Roche). Mitochondrial fractions were attained using differential centrifugation as performed previously [4]. Total protein content of cell lysates and mitochondrial fractions was determined using the BCA protein assay method [4].

### 2.3 Caspase activity

Enzymatic activity of caspase-2 and -3 was assessed using the fluorogenic substrates Ac-VDVAD-AMC and Ac-DEVD-AMC (Enzo Life Sciences) [4]. Cell lysates were loaded in duplicate at room temperature in black 96-well plates with the appropriate substrates. Fluorescence was measured using a SPECTRAmax Gemini XS microplate spectrofluorometer with excitation and emission wavelengths of 360 nm and 440 nm, respectively. Enzyme activity is expressed as fluorescence intensity in arbitrary units (AU) per milligram protein.

### 2.4 Immunoblotting

Lysed samples were further prepared in 4x SDS buffer (1.46 M sucrose, 7.5% SDS, 62.5 mM Tris–HCl (pH 6.8), 2 mM EDTA (pH 7.5), 200 mM DTT, and 1% bromophenol blue). Careful loading of vortexed samples of equal protein (30 µg) were separated by SDS/PAGE electrophoresis and then transferred onto PVDF membranes [4], [6]. Membranes were incubated with primary antibodies against myosin (MF20; 1:1000), myogenin (F5D; 1:200) (Developmental Studies Hybridoma Bank), actin (A2066; 1:2000) (Sigma Aldrich), MnSOD (ADI-SOD-110; 1:4000) (Enzo Life Sciences), or Drp1 (D6C7; 1:1000) (Cell Signaling) overnight at 4 °C and then with the appropriate horseradish peroxidase (HRP)-conjugated secondary antibody (Santa Cruz) for 1 h at room temperature. Bands were visualized using the Clarity ECL blotting substrate (Bio-Rad) and the ChemiGenius 2 Bio-Imaging System (Syngene). The approximate molecular weight for each protein was estimated using Precision Plus Protein WesternC Standards and Precision Protein Strep-Tactin HRP Conjugate (Bio-Rad). Equal loading and quality of transfer were confirmed by staining membranes with Ponceau S (Sigma Aldrich).

### 2.5 Fluorescent microscopy

Fluorescent microscopy was performed to determine changes in cellular and mitochondrial morphology in cells treated with STS and mdivi-1. After treatments, live cells were loaded with the dye MitoTracker Green AM (50 nM) for 30 min at 37 °C. Cells were then fixed with 4% formaldehyde and counterstained with DAPI for visualization of nuclei. Fluorescent microscopy was also used to visualize nuclei and myosin expression [4]. Cells grown on glass coverslips coated with gelatin were removed from culture at the indicated time points, fixed with 4% formaldehyde, and incubated with an anti-myosin antibody (MF20; Developmental Studies Hybridoma Bank) diluted in 10% goat serum in PBS for 1 h. Cells were then incubated with an Alexa Fluor 555-conjugated secondary antibody (ThermoFisher) for 1 h, stained with DAPI (ThermoFisher), and mounted with Prolong Gold Antifade Reagent (ThermoFisher). Images were captured with an Axio Observer Z1 fluorescent microscope equipped with standard Red/Green/Blue filters, an AxioCam HRm camera, and AxioVision software (Zeiss). Attained Images were used to calculate the degree of myoblast fusion by counting all cells in five random microscopic fields per experiment. Nuclei were categorized as follows: single-nucleated myosin negative (unfused), single-nucleated myosin positive (unfused with myosin), or multi-nucleated myosin positive (fused).

### 2.6 Statistics

Results are presented as means±SEM, where *n*=3 independent experiments conducted using different passages of cells. Comparisons were made using 1-way ANOVAs, with Tukey post-hoc analyses performed to detect differences between groups. For each analysis, *p*<0.05 was considered statistically significant.
